# Cardiovascular Imaging for the Primary Prevention of Atherosclerotic Cardiovascular Disease Events

**DOI:** 10.1007/s12410-015-9351-z

**Published:** 2015-08-14

**Authors:** Lauren A. Weber, Michael K. Cheezum, Jason M. Reese, Alison B. Lane, Ryan D. Haley, Meredith W. Lutz, Todd C. Villines

**Affiliations:** Cardiology Service, Walter Reed National Military Medical Center, 8901 Wisconsin Avenue, Bethesda, MD 20889 USA; Departments of Medicine and Radiology (Cardiovascular Division), Brigham and Women’s Hospital, Non-Invasive Cardiovascular Imaging Program, Boston, MA 02115 USA; Department of Medicine, Walter Reed National Military Medical Center, 8901 Wisconsin Avenue, Bethesda, MD 20889 USA

**Keywords:** Coronary artery calcium, Carotid intima-media thickness, Atherosclerosis, Cardiovascular risk, Primary prevention

## Abstract

Traditional cardiovascular risk factors have well-known limitations for the accurate assessment of individual cardiovascular risk. Unlike risk factor-based scores which rely on probabilistic calculations derived from population-based studies, coronary artery calcium (CAC) scoring, and carotid ultrasound allow for the direct visualization and quantification of subclinical atherosclerosis with the potential for a more accurate, personalized risk assessment and treatment approach. Among strategies used to guide preventive management, CAC scoring has consistently and convincingly outperformed traditional risk factors for the prediction of adverse cardiovascular events. Moreover, several studies have demonstrated the potential of CAC testing to improve precision for the use of more intensive pharmacologic therapies, such as aspirin and statins, in patients most likely to derive benefit, as compared to atherosclerotic cardiovascular disease risk calculators. By comparison to CAC, the role of carotid ultrasound for the measurement of carotid intima-media thickness (CIMT) remains less well-elucidated but may be significantly improved with the inclusion of plaque screening and novel three-dimensional measurements of plaque volume and morphology. Despite significant evidence supporting the ability of non-invasive atherosclerosis imaging (particularly CAC) to guide preventive management, imaging remains an under-utilized strategy among current guidelines and clinical practice. Herein, we review evidence regarding CAC and carotid ultrasound for patient risk classification, with a comparison of these techniques to currently advocated traditional risk factor-based scores.

## Introduction

Iterative changes in primary prevention guidelines resulting in increasingly intensive risk factor control have contributed to significant reductions in the rates of atherosclerotic cardiovascular disease (ASCVD) mortality. From 2001 to 2011, relative rates of coronary heart disease and stroke mortality declined by 30.8 and 35.1 %, respectfully [1]. Despite these remarkable reductions in cardiovascular mortality over the past several decades, atherosclerotic cardiovascular diseases remain the leading cause of death and preventable morbidity in the USA and other industrialized nations [1]. It is estimated that more than half of Americans will ultimately die of cardiovascular diseases, and nearly half of all acute coronary events occur in previously asymptomatic individuals [1]. Current guidelines and ASCVD primary prevention paradigms are based on the use of probabilistic risk scores that utilize a few standard cardiovascular risk factors to estimate future ASCVD risk [2••, 3••, 4••]. Widespread application of these population-derived, risk factor-based scores is attractive as they are simple, cheap, office-based and provide patients and providers with quantitative risk estimates over both a 10-year and longer term (30-year/lifetime) time horizon so to inform patient and provider behavior. However, it is important to recognize that there are numerous well-documented limitations to the widespread, sole reliance on traditional risk factor-based scores for the primary prevention of ASCVD in many individuals. These limitations, highlighted in part by controversy surrounding the 2013 American College of Cardiology/American Heart Association (ACC/AHA) Guideline on the Assessment of Cardiovascular Risk [2••], include concerns regarding imprecision in ASCVD risk estimation when applying scores used to predict population risk to an individual. For example, it is estimated that current pooled cohort risk scores may broadly overestimate ASCVD risk, potentially leading to large-scale overtreatment in individuals unlikely to benefit from long-term treatment with statins and aspirin [5–7], with potential for negative cost and clinical consequences [8]. Further, without considering proven risk factors such as family history of premature coronary heart disease, prior risk factor treatments (e.g., intensity and duration of prior statin use), the variability in risk factor measures (e.g., blood pressure), and the magnitude of prior risk factor elevations (e.g., prior but not current smoking), the use of “one-time” risk scores may significantly underestimate risk in some individuals, potentially resulting in under-treatment and less aggressive lifestyle modifications. Importantly, current risk scores have not been prospectively validated for their accuracy or ability to improve ASCVD outcomes and also lack validation among a number of prevalent, contemporary ethnicities.

Interest in individualized risk prediction has grown based on numerous studies involving diverse populations demonstrating that atherosclerotic imaging for identification and quantification of plaque burden more accurately identifies individual ASCVD risk as compared to risk scores and various biomarkers and may, therefore, better guide the application and intensity of preventative therapies [9–12]. Imaging has the potential to integrate lifetime exposure to measured and unmeasured (genetic, environmental) risk exposure and document manifest atherosclerosis, the strongest risk factor for future ASCVD events [13•]. As costs and radiation exposure have declined and guidelines for the management of incidental findings have matured with regards to atherosclerosis imaging, its use for more personalized ASCVD risk assessment has become increasingly attractive. Indeed, a central tenet in ASCVD primary prevention is that the use and intensity of preventative therapies should be matched to individual patient risk based on data demonstrating that the most intensive treatments are most effective when differentially applied to those at greatest ASCVD risk.

Herein, we review the literature regarding two of the most well-studied cardiovascular imaging techniques—coronary artery calcium scanning (CAC) and carotid ultrasound—for their use in the primary prevention of ASCVD events, highlighting current guideline recommendations as well as recommendations for implementation of these tests in patient management given limitations of current ASCVD risk scores.

## Limitations of Risk Factor-Based Scores for ASCVD Prediction and Treatment Decision-Making in Primary Prevention

The Framingham Risk Score (FRS) and similar risk factor-based scores rely most heavily on chronologic age and gender and are further refined through one-time measures of traditional modifiable cardiovascular risk factors, such as smoking status, blood pressure, cholesterol (total cholesterol and high-density lipoprotein cholesterol), and diabetic status. However, while the FRS is useful, it has been shown to only modestly predict incident coronary heart disease (CHD) events (coronary death and myocardial infarction (MI)), with a C-statistic of approximately 0.70 [6, 14].

Recently, the authors of the 2013 ACC/AHA guideline on the assessment of cardiovascular risk and the 2013 ACC/AHA guideline on the treatment of cholesterol derived a new risk calculator (pooled cohort risk equations) designed to estimate risk for both CHD and stroke (ASCVD) in white and black patients, while simultaneously reducing the risk threshold for the use of statin medications in patients without manifest ASCVD. In addition, the authors advocated for the use of longer-term risk calculators for the assessment of lifetime cardiovascular risk to potentially guide treatment decision-making in patients where statin medication were not clearly recommended by the 10-year ACC/AHA ASCVD (pooled cohort) equation. As stated above, the pooled cohort equations were recommended despite no prospective validation of their accuracy in contemporary populations and no studies documenting improved outcomes with their implementation. Moreover, several analyses in more contemporary populations than the derivation studies suggest that the pooled cohort equation may significantly overestimate ASCVD risk and the subsequent number of patients who would be likely to benefit from life-long statin therapy [5–7]. For example, comparison of the 2013 ACC/AHA ASCVD risk score, Framingham-based risk scores, and the Reynolds Risk Score (RRS) for the prediction of ASCVD events in 4967 Multi-Ethnic Study of Atherosclerosis (MESA) subjects followed over a median of 10.2 years demonstrated gross overestimation of risk by 37–154 % in men and 8–67 % in women, regardless of prior statin or aspirin treatment or subsequent coronary interventions [6]. In that study, among men with a 10-year ACC/AHA ASCVD risk score of 7.5–10 %, the observed event rate was only 3 %. Within this analysis, 49 % of subjects would have been stratified to moderate-high intensity statins, among which 41 % had a CAC (Agatston) score of 0, a group demonstrated repeatedly to have an exceedingly low event rate (5.2 events per 1000 patient years in this 10-year analysis). Similarly, potential under-treatment also occurred. For example, 5 % of patients not considered eligible for statin therapy had a CAC score >100, a group with high ASCVD risk. Overestimation of risk to variable degrees was demonstrated in several other modern primary prevention cohorts, demonstrating that the 2013 ACC/AHA ASCVD risk calculator only provides modest discriminatory predictive value (C-statistic ~0.7), particularly in white subjects and those previously treated with statins [5, 14, 15].

Not surprisingly, risk scores calculated at a single point in time may be inherently limited due to their inability to account for genetic influences, variability in measured risk factors (e.g., blood pressure) and prior, untreated risk factor severity and exposure duration. For example, prior smoking exposure is not considered in the current ACC/AHA ASCVD equation (only current smoking), ignoring potentially decades of exposure to one of the strongest ASCVD risk factors. Similarly, family history is also not considered in the 2013 ACC/AHA ASCVD risk score, with the clinical assessment of family history significantly downgraded as compared to prior risk assessment/primary prevention guidelines (from class I to IIb) [2••, 16]. Within the MESA study and other primary prevention cohorts, family history of premature CHD has been shown to be among the most powerful risk factors for the prediction of subsequent adverse cardiovascular events and is also strongly associated with more advanced subclinical atherosclerosis [17–19, 20••].

## The Evidence for Atherosclerosis Imaging to Improve ASCVD Risk Assessment and Primary Prevention

### Coronary Artery Calcium Scoring

**Fig. 1:**
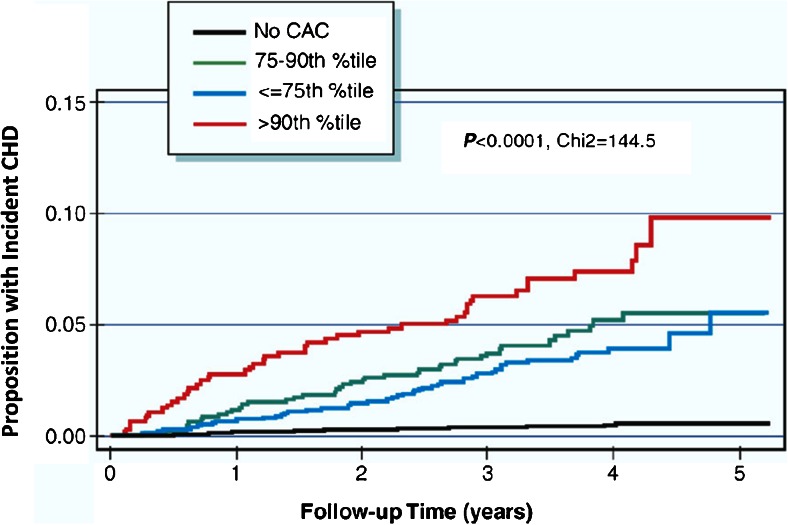
Kaplan-Meier curve for coronary heart disease events according to the coronary artery percentile score in the MESA (multi-ethnic study of atherosclerosis). *AC* coronary artery calcium, *CHD* coronary heart disease. Reproduced with permission from [23]

**Fig. 2:**
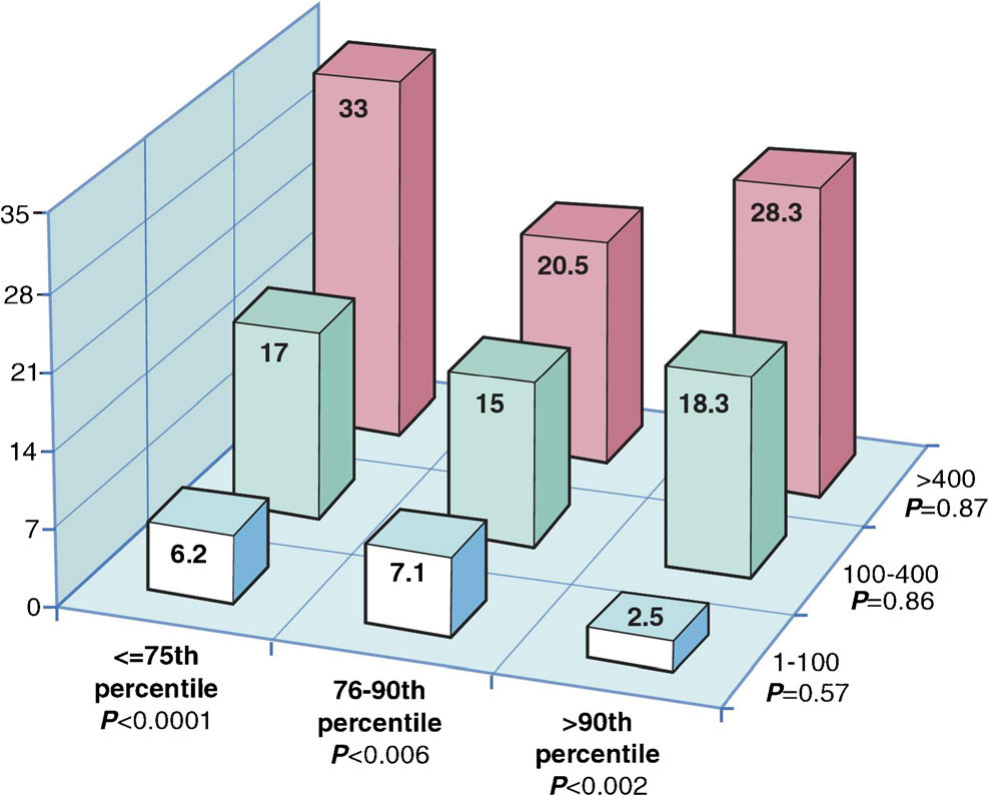
Rates of incident coronary heart disease per 1000 person years at risk by joint categories of the absolute coronary artery calcium group and age, sex, and race/ethnicity-specific percentiles. The rates of incident CHD per 1000 person years at risk by joint categories of the absolute CAC group and age-, sex-, and race/ethnicity-specific percentiles are displayed. Within a particular level of age, sex, and race/ethnicity-specific percentiles, there remains a clear trend of increasing risk across levels of the absolute CAC groups. In contrast, once the absolute CAC category is fixed, there is no increasing trend across levels of age, sex, and race/ethnicity-specific categories. *CAC* coronary artery calcium, *CHD* coronary heart disease. Reproduced with permission from [23]

Coronary artery calcium scanning is a rapid, non-contrast computed tomography (CT) of the heart used to identify calcification—defined as an area of ≥3 adjacent pixels (or 1 mm^2^) of at least 130 Hounsfield units—within epicardial coronary arteries. It is simple, can be performed in any patient with a single breath-hold and at very low-radiation exposure (≤1 mSv), comparable to radiation exposure associated with screening mammography [21, 22]. Coronary artery calcification is most commonly quantified using the Agatston method and is highly reproducible, resulting in the ability to categorize absolute scores as 0 (no CAC), 1–10 (minimal CAC), 11–100 (mild), 101–400 (moderate), >400 (severe), and >1000 (very severe). In addition to absolute scores, coronary atherosclerosis severity, as measured by CAC scoring, can be compared to asymptomatic subjects of the same age, gender, and ethnicity using established databases (e.g., MESA), resulting in calculation of an individual CAC percentile score, with both absolute and percentile scores used for cardiovascular risk refinement and medical decision-making (Figs. 1 and 2) [23].

### Prognostic Accuracy of Coronary Artery Calcium Scoring

Coronary artery calcium scoring has been repeatedly demonstrated to be the most robust predictor of coronary events in asymptomatic patients, particularly in patients estimated to be at intermediate risk based on risk factor scores. Without exception, every large-scale study performed to date has shown CAC to be superior to risk factor scores, with CAC resulting in significant reclassification of patients (Table 1). The MESA study, representing one of the most comprehensive prospective studies of CAC to date, is a large-scale (*n* = 6814) National Heart, Lung, and Blood Institute-sponsored study of CAC involving asymptomatic adult subjects (mean age 62 years) comprising four different ethnicities from the USA [10]. Initial study analysis performed at a mean of 3.8 years of follow-up for incident CHD confirmed earlier studies regarding the superiority of CAC over standard risk scores. Specifically, compared with patients that had a CAC score of 0, patients with CAC of 1–100 had a risk-adjusted hazard ratio of 3.61 for any cardiac event. For higher CAC scores, the hazards ratio increased incrementally to 7.73 and 9.67 for CAC scores of 101–300 and >300, respectively. Compared to standard risk variables, the addition of CAC markedly improved the accuracy of CHD event prediction, significantly increasing the area under the receiver-operating characteristic curve from 0.77 to 0.82 (*p* < 0.001). Further analysis demonstrated that, compared to the FRS, CAC resulted in significant net reclassification of individuals to more accurate risk categories [24]. For example, within MESA, the overall net reclassification index (NRI) of CAC over 5 years of follow-up was 25 % (meaning, 25 % of subjects were more accurately classified to a different risk category), with NRI values of 11.6, 54.4, and 35.0 % for FRS categories of 0–6 % (low risk), 6–20 % (low-intermediate risk), and >20 % (high risk), respectively. These findings were confirmed in several other large-scale prospective studies, demonstrating virtually identical NRI values (NRI 19–22 % overall), with NRI values of >50 % for patients at intermediate risk by the FRS [25, 26]. Follow-up of MESA participants extending to beyond 10 years (and up to 14 years) has confirmed the long-term prognostic value of CAC scoring [27–29].

**Table 1:** Prognostic power of coronary artery calcium in asymptomatic patients

First author [Ref. #]	N	Mean age (years)	Follow-up (years)	Agatston calcium score cutoff	Comparator group for relative risk calculation	Relative risk ratio
Arad et al. [9]	1173	53	3.6	>160	<160	20.2
Wong et al. [63]	926	54	3.3	Top quartile (>270)	First quartile	8.8
Greenland et al. [16]	1312	66	7.0	>300	No CAC	3.9
Shaw et al. [46•]	10,377	53	5	≥400	≤10	8.4
Arad et al. [34]	5585	59	4.3	≥100	<100	10.7
Taylor et al. [12]	2000	40–50	3.0	>44	0	11.8
Vliegenthart et al. [64]	1795	71	3.3	>1000	<100	8.3
				400–1000	<100	4.6
Budoff et al. [46•]	25,503	56	6.8	>400	0	9.2
Lakoski et al. [65]	3601	45–84	3.75	>0	0	6.5
Becker et al. [66]	1726	57.7	3.4	>400	0	6.8 men
						7.9 women
Detrano et al. [10]	6814	62.2	3.8	>300	0	14.1
Erbel et al. [26]	4487	45–75	5	>75th percentile	<25th percentile	11.1 men
						3.2 women

One of the criticisms of CAC scoring raised by the authors of the 2013 ACC/AHA risk assessment guidelines is the focus on CHD outcomes by most CAC studies, with little data regarding the prognostic value of CAC for the combined outcome of ASCVD; the endpoint estimated by the current ACC/AHA ASCVD calculator. Given the impressive net reclassification demonstrated by CAC scoring and the population clinical and cost burden of CHD as a critical part of ASCVD, this criticism appeared to be shortsighted, particularly given limitations of risk scores. Investigators from the MESA study recently demonstrated that CAC scoring was also superior to standard cardiovascular and stroke risk factors for the prediction of stroke and overall ASCVD [6, 30], similar to findings from the Heinz Nixdorf Recall Study [31]. In addition, CAC testing appears to be superior to long-term risk scores (lifetime risk scores) for the prediction of CHD events [32].

**Fig. 3:**
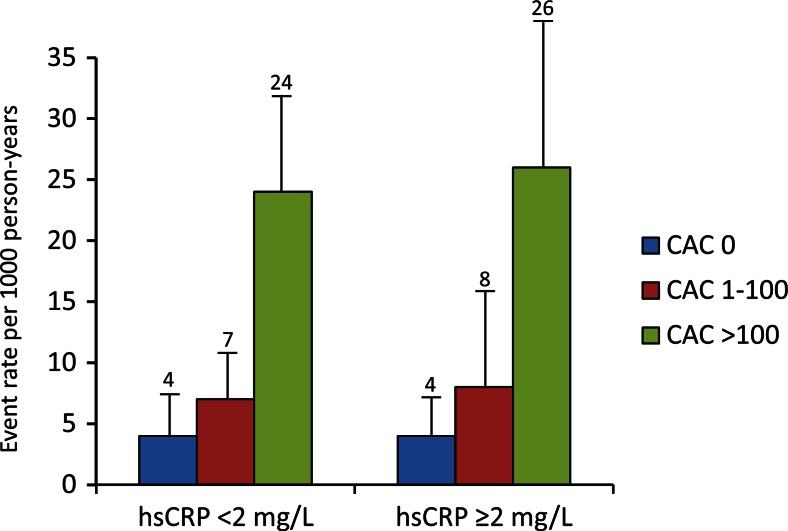
Relationship of high-sensitivity C-reactive protein to coronary artery calcium score and coronary heart disease event rates in MESA. *MESA* multi-ethnic study of atherosclerosis. Reproduced with permission from [35•]

The use of biomarkers, such as high-sensitivity C-reactive protein (hs-CRP), has also been explored as a potential approach to further improve risk factor-based approaches to ASCVD primary prevention, as with the Reynold’s Risk Score [14, 33]. Current risk assessment guidelines discuss hs-CRP as a possible tool in select patients [2••]. However, numerous studies comparing CAC to hs-CRP have repeatedly demonstrated it to be inferior to CAC for cardiovascular event prediction, noting that it poorly correlates with coronary atherosclerotic disease burden and demonstrates significant intra-individual variability on repeat testing, limiting its usefulness and attractiveness as a one-time biomarker utilized to base life-long treatment and risk assessments [20••, 34, 35•, 36, 37]. As one example, among 706 individuals in MESA, 69 % with a hs-CRP >3.0 mg/L (high risk category) were subsequently noted to have a discordant hs-CRP level in a low-risk category on repeat testing, with hs-CRP demonstrating greater intra-individual variability than standard cholesterol levels utilized for risk scoring [38]. The MESA investigators attempted to assess the role of hs-CRP using the Justification for the Use of Statin in Prevention: An Intervention Trial Evaluating Rosuvastatin (JUPITER) trial inclusion criteria (hs-CRP >2 and low-density lipoprotein <130 mg/dl) and matched them to MESA subjects with hs-CRP <2 [35•]. They found no effect of hs-CRP on outcomes and no relationship of hs-CRP to CAC (Fig. 3). Additionally, based on expected event rate reduction from statins, estimated number needed to treat (NNT) for rosuvastatin was 549, 94, and 24 for CAC score of 0, 1–100, and >100, respectively. Studies assessing the use of multiple biomarkers added to standard risk factor-based scores have similarly shown inferior performance as compared to direct measurement of coronary atherosclerotic burden using CAC [39].

### Power of Zero and Impact of CAC on Cost, Treatment, and Outcomes

One of the most consistent findings across all studies of CAC is the impressively low 10-year cardiovascular risk (approximately 1 %) observed in subjects with CAC = 0, regardless of risk factor status [40, 41]. Given concerns about overestimation of ASCVD risk and subsequent overtreatment, calcium scoring provides the potential to focus preventative treatments more appropriately, while avoiding unnecessary treatment in patients at very low ASCVD risk (CAC = 0). While CAC testing is generally cheap to perform (≤$100), many have raised concerns about increased costs induced from the utilization of CAC scoring due to increased subsequent downstream cardiovascular testing [42]. In the Early Identification of Subclinical Atherosclerosis by Non-invasive Imaging Research (EISNER) study, CAC testing was compared to usual care in asymptomatic adults without baseline ASCVD for the primary outcome of change in calculated cardiovascular risk [43, 44]. In an EISNER cohort of 1381 subjects, additional cardiovascular testing was differentially performed according to CAC severity: infrequent in patients with minimal or no CAC (CAC<10) but more frequently among participants with CAC scores ≥400. Similarly, the rate of invasive coronary angiography and coronary revascularizations at 1-year were higher in those with severely elevated CAC scores (19 % of subjects with a CAC score ≥1000). However, the performance of CAC testing did not significantly increase overall healthcare costs as the absence of CAC was associated with significantly lower rates of subsequent cardiovascular testing and costs. CAC scoring was also associated with overall significant improvements in several cardiovascular risk factors (blood pressure, low-density lipoprotein cholesterol, and waist circumference), with findings similar to other studies demonstrating increased adherence to primary prevention medications among patients with prevalent CAC [45]. In addition, an analysis of the MESA demonstrated the importance of appropriate patient selection and risk of overtreatment in the application of primary preventative therapies. For example, among subjects with CAC = 0, the use of aspirin was estimated to result in net harm, while the application of aspirin for primary prevention in patients with CAC ≥100 (at higher risk) was demonstrated to be effective in a net clinical benefit analysis [8].

Unfortunately, there are no prospective, randomized outcomes trials assessing the impact of CAC testing on long-term outcomes. St. Francis Heart Study investigators randomized 1005 with severely elevated CAC (>80th percentile) asymptomatic subjects to atorvastatin 20 mg daily or placebo and reported a significant reduction (15.0 vs. 8.7 %; *p* = 0.046) in major adverse cardiovascular events (inclusive of coronary and peripheral revascularization) over 4.3 years of follow-up among those with a baseline CAC score >400 treated with atorvastatin. Unfortunately, further large-scale studies have not yet been performed, likely due to cost concerns related to the expected size and duration of such a study [46•].

## The Evidence for Atherosclerosis Imaging to Improve ASCVD Risk Assessment and Primary Prevention

### Carotid Intima-Media Thickness and Plaque Screening

Ultrasonography for the measurement of carotid artery intima-media thickness (CIMT) and carotid plaque in asymptomatic screening populations has been extensively studied and well-described. Compared to CAC, the use of ultrasonography for the detection of subclinical atherosclerosis for primary ASCVD risk prevention purposes is attractive as it requires no ionizing radiation, may result in few incidental findings, is highly reproducible, and can typically be done in an office setting with appropriately trained personnel. Furthermore, carotid ultrasonography can be performed using equipment and software often already available to patients and clinicians.

Current measurement techniques, aided by modern high-frequency ultrasound equipment and semi-automatic measurement software, are well delineated in current guideline statements and have been shown to be highly reproducible among trained sonographers [47, 48]. While performance of carotid ultrasonography is not the focus of this review, the American Society of Echocardiography (ASE) recommends that CIMT be measured using ultrasound images from the distal 1 cm of the far wall of each common carotid artery (CCA), as measurements involving the near wall are often suboptimal, but also that CIMT measurement be supplemented with a carotid plaque-screening scan of the full extracranial carotid arterial system [47]. Specifically, this plaque screen involves obtaining circumferential scans of the CCA, carotid bulb, and internal and external carotid arteries. The actual CIMT measurement is obtained by tracing the blood-intima and media-adventitia boundaries along a 1-cm length of the CCA far wall using a leading-edge to leading-edge technique. Automated border detection programs can be used to reduce measurement variability when performing CIMT measurements.

Carotid plaque is defined by the ASE as the presence of focal wall thickening that is at least 50 % greater than that of the surrounding vessel wall or as a focal region with CIMT >1.5 mm that protrudes into the lumen that is distinct from the adjacent boundary. Plaque measurements (e.g., volume or morphology) further characterized outside of either the presence or absence of plaque has not been well studied and thus far has not been incorporated in imaging interpretation. The European Society of Cardiology (ESC) definition of carotid plaque differs slightly. The ESC defines CIMT of >0.9 mm as abnormal and plaque as any focal structure of the inner vessel wall measuring at least 0.5 mm or greater or any IMT measurement greater than or equal to 1.5 mm [3••].

### Prognostic Accuracy of CIMT and Plaque by Carotid Ultrasound

The major studies assessing the prognostic value of CIMT are summarized in Table 2. It is important to note when considering the evidence base that there is significant heterogeneity across studies with regards to the site of CIMT and plaque assessments. For example, many studies included both near and far wall measurements in their data sets and not all included plaque screening. With inconsistency in the acquisition of CIMT values, so too is the inconsistency in reported values. Measurements are usually reported as an average of all measurements, mean maximum (average of the maximum value for all segments), or individual maximum values from any segment. Carotid plaque definitions also varied, with reporting as present or present and characterized by risk (low, intermediate, or high risk plaque) without further specification. Some studies include plaque measurements as part of CIMT, consistent with ASE recommendations, although this was not uniform. As such, attempts to derive consensus on the use of CIMT for risk prediction have suffered from significant heterogeneity between studies.

**Table 2:** ᅟ

Study [Reference #]	Sample Size	Age of Subjects	Follow-up	Carotid Ultrasound Parameters	Plaque	Endpoints	CIMT, RR (95% CI)	NRI
APSIS [67]	558	60 + 7 yrs	Median, 3.0 yrs	Max left CCA-IMT, far wall	Not specified	CV death, MI, revascularization	IMT>1.02mm, RR: 0.78 (0.36-1.70) for CV death or MI; RR: 1.07 (0.56-2.04) for revascularization	
ARIC [68]	12,841	45-64 yrs	Mean follow-up 15.1 yrs	Mean far wall IMT at 6 sites (CCA, bulb, ICA, bilateral)	Plaque included	MI, CV death	IMT > 1.0mm: women HR: 5.07 (3.08-8.36); men 1.85 (1.28-2.69)	7.1
CAPS [69]	5,056	19-90yrs	Mean follow-up 4.2 yrs	Mean far wall IMT bilaterally at CCA, carotid BIF, ICA bulb	Not specified	MI, stroke, death	RR for 1 SD: RR 1.17(1.08-1.26) for CCA-IMT; RR 1.14 (1.05-1.24), for carotid bulb-IMT; RR 1.09 (1.01-1.18) for ICA-IMT.	-1.4
CCCC [70]	2,190	> 35 yrs	Median, 10.5 yrs	Maximal CCA-IMT, far wall, bilateral	Plaque excluded	MI, CV death, PCI, CABG	RR: 1SD; 1.38 (1.12-1.70)	
Charlottesville Study [71]	727	16-85 yrs	Mean, 4.78 yrs	Mean CCA-IMT, bulb-IMT, ICA-IMT, near and far wall bilaterally	Plaque included	MI, revascularization, stroke, TIA	OR for highest quartile of carotid bulb IMT: 5.8 (1.3-26.6)	
CHS [72]	5,020	72.6+5.5 yrs	5 days to 12 yrs (median, 11 yrs)	CCA and ICA-IMT, mean of maximal IMT, near and far wall bilaterally	Plaque included	MI, stroke, CV death, all-cause mortality	Highest tertile: RR: 1.84 (1.54-2.20)	
Cournot, et al. [73]	2,561	51.6 + 10.5 yrs	2-10 yrs	CCA-IMT, ICA-IMT bilaterally	Plaque excluded	CV death, MI, angina	IMT >0.63mm; HR: 2.26 (1.35-3.79)	
FATE [74]	1,574	49.4 + 9.9yrs	Mean, 7.2 yrs	Right CCA-IMT	Plaque excluded	CV death, revacularization, MI, angina, stroke	HR: 1.45 (1.15-1.83)	11.6%
Framingham Offspring Study [75]	2,965	58 + 10yrs	Average, 7.2yrs	Mean CCA-IMT, or maximal CCA-IMT, maximal ICA-IMT, bilaterally	Plaque excluded	MI, angina, CV death, stroke, claudication, heart failure	HR for 1-SD mean CCA-IMT: 1.13 (1.02-1.24); HR for 1-SD maximal CCA-IMT: 1.21 (1.13-1.29); HR for 1-SD maximal ICA-IMT: 1.21 (1.13-1.29)	CCA: O% ICA: 7.6%
IMPROVE [76]	3,703	Median 64.4yrs	Median 36.2 months	Maximal and mean CCA, ICA, BIF, bilaterally	Plaque included	MI, SCD, angina, stoke, TIA, heart failure, revascularization	HR for 1-SD increase: mean CCA-IMT: 1.33 (1.18-1.50); mean BIF-IMT: 1.28 (1.12-1.47); mean ICA-IMT: 1.34 (1.18-1.51)	FRF+ICCAD+IMT mean-max 12.1%
KIHD [77]	1,257	42-60 yrs	1 mo- 2.5yrs	CCA-IMT, mean of max IMT, near and far wall bilaterally	Focal calcified plaque not included	AMI	CCA-IMT increment, 0.1mm; RR: 2.14 (1.08-4.26)	
LILAC [78]	298	Mean, 79.6yrs	Mean 1,152 days	Average of CCA bilaterally, near and fall wall	Not specified	All-cause mortality	For 0.3mm increase in left IMT, RR: 1.65 (1.08-2.5); right IMT, RR: 3.3 (1.4-1.7)	
MESA [20]	6,814	45-84 yrs	Median, 7.6yrs	Mean of max right CCA-IMT, far wall	Plaque excluded	MI, revascularization, SCD, CV death	HR: 1.17 (0.95-1.45)	Mean-max IMT 7.0% Max-IMT 6.8%
MDCS [79]	5,163	46-68 yrs	Median 7yrs	Mean far wall right distal CCA	Plaque included	MI, CV death	RR for highest tertile: 1.50 (0.81-2.59)	
OSACA2 [80]	574	65.3 + 9.5yrs	Mean, 2.6yrs	Mean maximal CCA-IMT, BIF-IMT, ICA-IMT, near and far wall bilaterally	Plaque included	MI, CABG, angioplasty, PAD, stroke	For 1-SD increase, RR: 1.57 (1.11-2.20)	
Rotterdam Study [81]	6.389	69.3 + 9.2 yrs	7-10 years	Avg of max CCA-IMT or near and far wall bilaterally	Not specified	MI	RR: 1.95 (1.19-3.19)	CAD, Stroke Men: 0.2, 3.9 Women: 8.2, 8.0
The Edinburg Artery Study [82]	1,007	Mean 69.4 yrs	12 years	Max far wall CCA-IMT bilaterally	Not specified	MI, stroke, angina, claudication	IMT > 0.9mm, OR: 1.59 (1.07-2.37)	
Three-City Study [83]	5,895	65-85 yrs	Median 5.4yrs	Mean CCA-IMT bilaterally, near and far wall	Plaque measured separately	MI, angina, CV death, revascularization	HR for fifth quintile: 0.8 (0.5-1.2)	Carotid plaque 13.7%
Tromso Study [84]	6,226	25-84yrs	6 years	Mean of near and far wall right CCA-IMT and far wall of the bulb	Plaque included	MI	Highest IMT quartile, 1.73 (0.98-3.06) in men and 2.86 (1.07-7.65) in women	

Notwithstanding the heterogeneity in CIMT measurements across studies, viewed in its entirety and considering the best available evidence, it appears that increasing CIMT is associated with an independent increase in ASCVD events. However, the ability of CIMT to predict adverse events as compared to risk factors, without consideration of carotid plaque, is less robust. For example, the NRI using CIMT alone from current studies (Table 2) has been shown to be minimal, ranging from 0 to 12 %. Notwithstanding issues with study population and measurement heterogeneity, it is important to note that the measurement of CIMT and plaque may represent variations of atherosclerotic pathophysiology. For example, while CIMT represents the thickness of the intima-media, an area that increases both as a factor of aging and smooth muscle hypertrophy, plaque is specific for overt atherosclerosis, a process resulting from the deposition of foam cells, smooth muscle cells, macrophages, lipid core, and a fibrous cap [47, 49•]. Hence, subjects with overt plaque may represent a distinctly higher risk population.

**Fig. 4:**
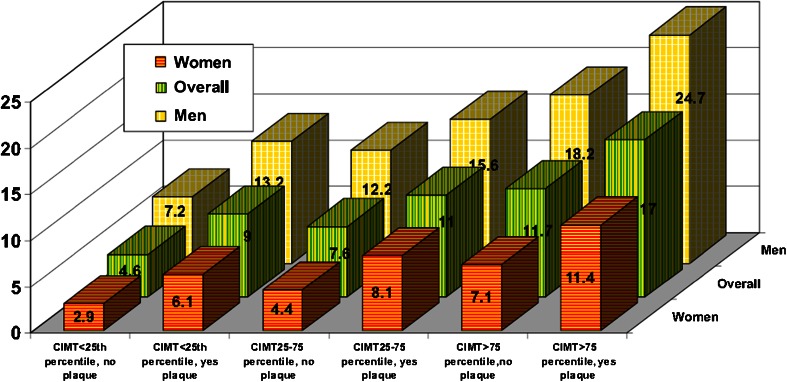
Adjusted coronary heart disease incidence rate per 1000 person years adjusted by CIMT categories with and without plaque. For every carotid intima-media thickness (CIMT) category (i.e., <25th percentile, 25th to 75th percentile, and >75th percentile), for the overall group (*green bars*), men (*yellow bars*), or women (*orange bars*), having carotid artery plaque is associated with a higher incidence of coronary heart disease. Reproduced with permission from [11]

In the Atherosclerosis Risk in Communities study (ARIC) of 13,145 health subjects between 45 and 64 years of age at the time of baseline carotid ultrasonography, the best model to predict incident cardiovascular events (during mean follow-up of 15.2 years) included traditional risk factors, CIMT, and plaque (Fig. 4) [11]. The NRI for CIMT was 9.9 %; however, the addition of plaque to any level of CIMT (<25th percentile, 25–75th percentile or >75th percentile) significantly improved risk prediction in men and particularly among women. A meta-analysis of 11 population-based studies (*n* = 54,336) confirmed the ARIC findings, concluding that carotid plaque, when compared to CIMT alone (inclusive of various sites of measurement), had significantly higher diagnostic accuracy for incident myocardial infarction [50]. Similarly, meta-analyses limited to only CIMT versus those including plaque, despite several common authors, have reached opposite conclusions regarding the role of carotid ultrasonography for the prediction of ASCVD events. Across studies incorporating plaque, the NRI of carotid ultrasonography is ~8–11 %, with more robust results for CIMT and carotid plaque among studies utilizing patient level data (the most accurate method for performing a meta-analysis) [51, 52].

The improvement in risk prediction with inclusion of carotid plaque may not be surprising. In addition to potential differences in pathology between CIMT and plaque, overt plaque tends to form at the carotid bulb and internal carotid artery, areas often excluded from CIMT measurements. Hence, the exclusion of plaque from many CIMT studies may explain the less robust predictive value of CIMT alone. More recently, studies have attempted to further refine plaque assessment, moving from binary plaque classification (present vs. absent) to more quantitative and qualitative descriptions using 3-dimensional imaging techniques [53, 54]. It remains to be seen whether the assessment of carotid plaque area, volume, and plaque characteristics assessed using modern 3-dimensional measurements, such as surface irregularity, echolucency, and plaque texture, can further improve risk stratification. Interestingly, 3-dimensional assessment of plaque volume does appear to correlate more strongly with CAC scoring than CIMT, highlighting the promise of this technology [54].

## CAC Versus CIMT and Carotid Plaque

Several observations can be made from the studies that have compared the prognostic performance of CAC and carotid ultrasonography. First, CAC appears to be significantly more powerful than CIMT for the prediction of CHD events. Specifically, the NRI for CAC is consistently >20 % for CHD events and reaches >50 % among intermediate risk individuals, as compared to NRI values typically <10 % for carotid plaque measures. In the MESA study, CAC was superior to CIMT (without plaque assessment), hs-CRP, family history, and other novel risk markers for incident CHD event prediction [20••] and was superior to CIMT (without plaque assessed) for stroke prediction in the Heinz Nixdorf Recall study [55]. However, CIMT measurements may be comparable to CAC for the prediction of ASCVD events in elderly subjects [56, 57]. Finally, carotid ultrasonography may be more sensitive for the detection of early, subclinical atherosclerosis, particularly in younger patients without CAC [58]. The prognostic implications of this finding remain to be elucidated, particularly given the known excellent prognosis in patients with CAC = 0.

## Guideline and Appropriate Use Recommendations for CAC and Carotid Ultrasound

### Coronary Artery Calcium Scoring

With few exceptions, CAC testing is often recommended for use in asymptomatic patients at intermediate risk for CHD by standard risk assessment (Table 3). The 2013 ACC/AHA guidelines on cardiovascular risk assessment and cholesterol treatment suggest that CAC scoring may be reasonable (IIb recommendation) for further risk assessment in patients with a 5 to 7.5 % 10-year ASCVD risk [2••, 4••]. Within this low-intermediate risk group, the guideline writers suggest a threshold (based on expert opinion) of 300 Agatston units or 75th percentile (for age, sex, and ethnicity), above which risk assessment should be revised upward, potentially influencing decision to initiate statin treatment. Of note, this is a significant change from the 2010 guidelines which considered CAC testing and CIMT testing as reasonable to perform (IIa recommendation) in patients at intermediate risk (10–20 % FRS) [16]. The 2012 practice guidelines from the European Society of Cardiology support a similar approach, recommending that CAC should be considered in asymptomatic patients at moderate risk via traditional risk factor assessment (10-year CVD risk between 1 and 5 % by the SCORE calculator) [3••]. Similarly, the Canadian Cardiovascular Society recommends consideration of secondary non-invasive ASCVD testing (including CAC score) for intermediate-risk patients and further specifies that a statin should be started when CAC exceeds 100 [59]. Of note, current appropriate use criteria consider CAC testing appropriate in a number of patient cohorts. For example, the 2010 ACC/AHA appropriate use criteria for cardiac CT consider CAC appropriate in patients at intermediate CHD risk (10–20 % by FRS), and those at low risk (<10 % FRS) with a family history of premature CHD [60]. Similarly, the 2014 American College of Radiology (ACR) Appropriateness Criteria for CAD recommending CAC as a complementary risk stratification tool that is “usually appropriate” in intermediate risk patients and “may be appropriate” in low-risk patients with a family history of early CHD [61]. By comparison to older recommendations, the US Preventive Services Task Force found insufficient evidence in 2009 to recommend CAC testing in asymptomatic patients with no known history of CVD, citing the need for additional data on utility and cost-effectiveness (even in patients at intermediate risk by traditional risk assessment methods) [62].

**Table 3:**
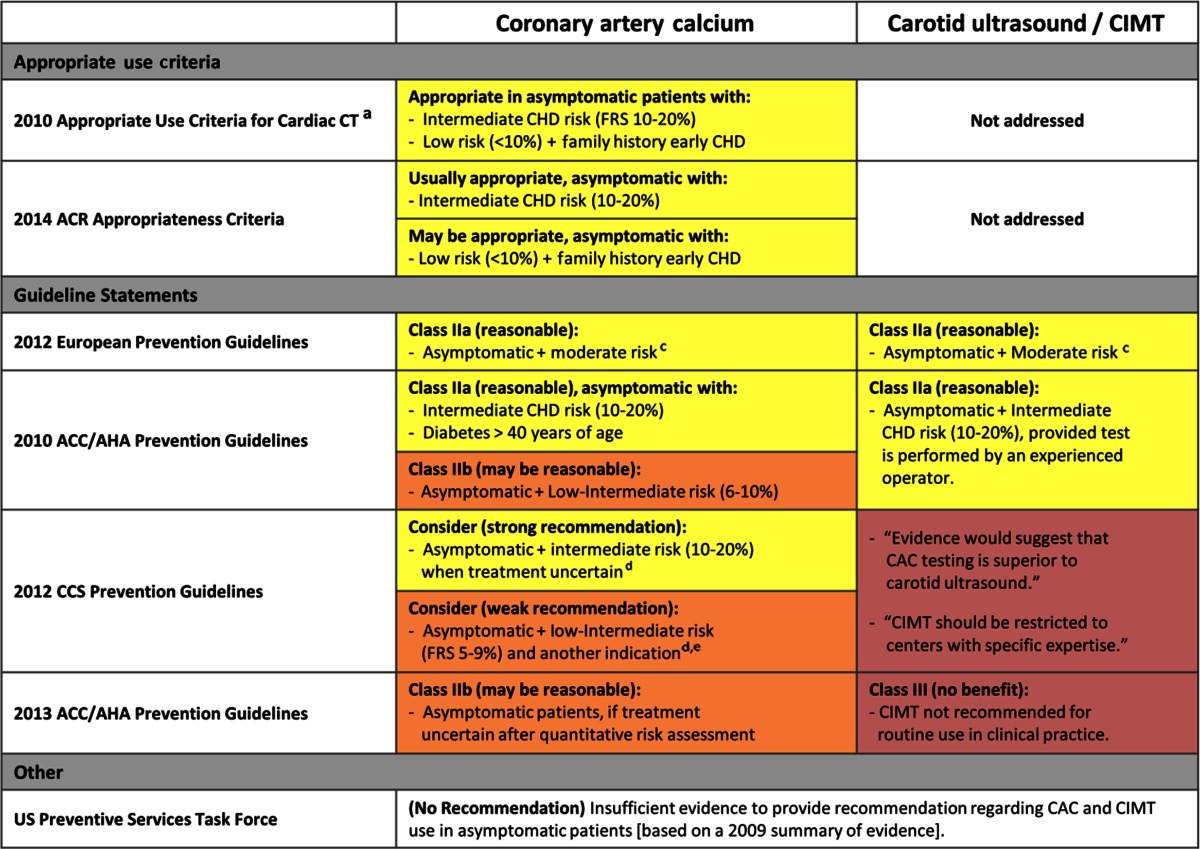
Recommendations for CAC testing and carotid ultrasound

*CAC* coronary artery calcium, *CAD* coronary artery disease, *CHD* coronary heart disease, *CIMT* carotid intima-media thickness, *FRS* Framingham Risk Score, *SIHD* stable ischemic heart disease

^a^Endorsed by ACCF/SCCT/ACR/AHA/ASE/ASNC/NASCI/SCAI/SCMR

^b^Endorsed by ACCF/AHA/ACP/AATS/PCNA/SCAI/STS

^c^ESC guidelines utilize the Systematic Coronary Risk Evaluation Project (SCORE calculator), where moderate risk is ≥1 and <5 % risk of fatal CVD at 10 years

^d^CCS guidelines state CAC superior to CIMT, and argues to consider CAC among secondary tests, but qualifies need for further data before CAC can be widely advocated

^e^For whom further risk assessment is indicated (e.g., strong family history of premature CAD, abdominal obesity, South Asian ancestry, or impaired glucose tolerance)

### Carotid Intima-Media Thickness

By comparison to CAC testing, recommendations for carotid intima-media thickness (CIMT) use vary considerably (Table 3). The 2013 ACC/AHA guidelines state that CIMT should not be routinely performed (Class III; no benefit), regardless of initial risk stratification by traditional methods [2••]. Of note, carotid plaque screening was not discussed. In contrast, the 2012 European Society of Cardiology and 2010 ACC/AHA guidelines recommend CIMT use (Class IIa; reasonable) for further evaluation of moderate or intermediate risk patients [3••, 59]. Despite variable recommendations for CIMT use, the guidelines agree that when CIMT is performed, it should be performed by an experienced operator to ensure a consistent approach and meaningful interpretation of findings across centers. Similar to CAC, CIMT was not endorsed by the USPTF citing the need for additional outcomes studies [62].

## Conclusions

Despite a significant decline in the rate of death from ASCVD over the past several decades, cardiovascular diseases remain the most common cause of morbidity and mortality in most countries. While efforts to refine traditional risk factor-based risk scores to more accurately predict ASCVD among several ethnicities were admirable, we feel that sole reliance on current ASCVD risk scores performed at a single point in time may be a fundamentally suboptimal, particularly when making life-long treatment decision in asymptomatic patients. Non-invasive imaging for the detection and quantification of subclinical atherosclerosis, particularly utilizing low-radiation CAC testing, has been definitely proven to be superior to risk factors-based scores for prognostic accuracy, especially in patients at intermediate risk according to risk scores or those with a family history of early cardiovascular disease. As data continues to mature for both CAC and carotid plaque imaging, public acceptance of the status quo with regards to the limitations of current risk factor-based prevention strategies, combined with physician awareness, may lead to increased appropriate use of imaging to more effectively prevent ASCVD.
